# Development of Low-Viscosity and High-Performance Biobased Monobenzoxazine from Tyrosol and Furfurylamine

**DOI:** 10.3390/ma14020440

**Published:** 2021-01-18

**Authors:** Zhibin Wen, Leïla Bonnaud, Rosica Mincheva, Philippe Dubois, Jean-Marie Raquez

**Affiliations:** 1 Shenzhen Institute of Advanced Electronic Materials, Shenzhen Institutes of Advanced Technology, Chinese Academy of Sciences, Shenzhen 518055, China; 2 Laboratory of Polymeric and Composite Materials Center of Innovation and Research in Materials and Polymers, Materia Nova Research Center and University of Mons, 23 Place du Parc, B-7000 Mons, Belgium; rosica.mincheva@umons.ac.be (R.M.); philippe.dubois@umons.ac.be (P.D.)

**Keywords:** high performance, fully biobased, tyrosol, polybenzoxazine

## Abstract

This work details the scalable and solventless synthesis of a potential fully biobased monobenzoxazine resin derived from tyrosol and furfurylamine. The structure of the monomer was studied by nuclear magnetic resonance (NMR) spectroscopy and Fourier transform infrared (FTIR). The curing of the precursors was characterized by differential scanning calorimetry (DSC), rheological measurements, and thermogravimetric analysis (TGA). The properties of the resulting biobased polybenzoxazine were then determined by thermogravimetric analysis (TGA) and dynamic mechanical thermal analysis (DMA). A thermally stable resin was obtained with 5% and 10% weight-reduction-temperature (*T*_d5_ and *T*_d10_) values of 349 and 395 °C, respectively, and a char yield of 53%. Moreover, the low melting temperature, low viscosity, and excellent thermomechanical behavior make this fully biobased resin a promising candidate for coating applications.

## 1. Introduction

The interest for polybenzoxazines from both academia and industry has been rising and has intensified in the past few decades. This class of materials has many unique characteristics such as good mechanical and thermal properties, excellent chemical resistance, low water uptake, and near-zero shrinkage, which make them potentially suitable for practical applications such as in the aerospace, electronic-packaging, and transportation industries [1,2,3].

Polybenzoxazines are usually obtained through a ring-opening polymerization process of heterocyclic six-membered 1,3-benzoxazine monomer most commonly synthesized by a Mannich-like condensation of a phenol, a primary amine, and formaldehyde [3]. The design flexibility results in numerous novel constantly increasing structures of monomers; however, traditional and unfunctionalized benzoxazine resins demonstrated high curing temperatures (typically 250 °C) that limit their use in practical applications [4]. Compared to traditional benzoxazine resins, hydroxyl monomers draw much attention for their contribution to low polymerization temperatures [5,6]. Kiskan et al. synthesized a novel with more processible fluid benzoxazine monomers bearing hydroxyethyl functions. A lower polymerization temperature of their benzoxazine monomers was reported compared to that of unfunctionalized ones. This low curing temperature was attributed to the presence of the hydroxyl groups to provoke the favorable alignment of monomers in the liquid state [7]. Kudoh et al. subsequently studied the action mechanism of hydroxyl groups and their functions in the ring-opening polymerization of benzoxazine function. Because of the intramolecular reaction between the hydroxyl group and zwitterionic intermediates, the hydroxyethyl benzoxazine exhibited much faster polymerized speed than that of similarly unsubstituted N-alkyl monomers [8]. Baqar et al. also demonstrated that intramolecular hydrogen bonding in presence of benzoxazines containing hydroxyl groups accelerated the ring-opening polymerization [9].

Nowadays, facing the possibility of oil shortages and the increase in CO_2_ emissions, the replacement of petrobased phenolic or amine compounds by similar derivatives issued from renewable and sustainable resources is gaining considerable attention. More precisely, a large number of natural phenol derivatives such as cardanol [10], vanillin, [11], eugenol [12,13,14], chavicol [15], guaiacol [16], sesamol [17], umbelliferone [18], arbutin [19], urushiol [20], catechol [21], and paracoumaric acid [22] were tested as potentially renewable raw materials for benzoxazine precursors synthesis. In addition, furfurylamine was chosen as the amine source because of its intrinsic valuable properties: (1) the furan ring participates in polymerization as additional crosslinking reaction to enhance the crosslink density and thermal resistance of the resulting resins [23]; (2) the cooperative activation effect of furan could lower the ring-opening polymerization temperature. This effect was studied by Froimowicz et al. with a comparative experiment between a coumarin-containing benzoxazine U–fa (synthesized from umbelliferone and furfurylamine) and U–a (made from umbelliferone and aniline). They reported that the furfurylamine-based benzoxazine decreased the polymerization temperature by about 22 °C [24].

Moreover, the obtention of thin layers is rather difficult with the high melting point of benzoxazine powder, and a solvent is usually required. In order to develop a solventless approach, low melting temperature and low viscosity combined with excellent performance materials are desired [22,25]. For instance, Thirukumaran et al. reported a cardanol-based benzoxazine showing low melting temperature; however, the material presented poor properties [10]. Sini et al. synthesized a V–fa monomer from vanillin and furfurylamine exhibiting a high *T*_g_ at 270 °C, but melting temperature was as high as 125 °C, which significantly affected the processability [25]. In the present work, a potentially fully biobased monobenzoxazine resin was synthesized from tyrosol, furfurylamine, and paraformaldehyde. A natural paraethanol phenol, tyrosol, which presents in a variety of natural sources, was chosen as hydroxylated phenol derivative to lower polymerization temperature. Meanwhile, the amine source furfurylamine was employed to increase crosslinking density and lower polymerization. The chemical structure of the precursors was studied by Fourier transform infrared (FTIR) and nuclear magnetic resonance (NMR) spectroscopy. Their curing characteristics were determined by FTIR, differential scanning calorimetry (DSC), and rheological measurements. After curing, the properties of the resulting biobased polybenzoxazine systems were characterized by thermogravimetric analysis (TGA) and dynamic mechanical thermal analysis (DMA). The low melting temperature, low viscosity, and excellent thermomechanical behavior make this fully biobased monobenzoxazine a promising candidate for practical application, such as anticorrosion coatings on aluminum.

## 2. Materials and Methods

### 2.1. Materials

Tyrosol (99%), furfurylamine (99%), and paraformaldehyde (95%) were purchased from Sigma-Aldrich. All chemicals were used directly without any further purification.

### 2.2. Characterization and Measurements

Nuclear magnetic resonance (NMR): Structures of precursors were determined by ^1^H NMR recorded with an NMR spectrometer (Bruker, 500 MHz) at room temperature. Deuterated chloroform (CDCl_3_) was chosen as the solvent and tetramethyl silane (TMS) as the internal reference.

Fourier transform infrared spectroscopy (FTIR): Precursors and crosslinked polymers were analyzed using a Bruker IFS 66v/S spectrometer to detect the conversions of benzoxazine and furan rings. Record range: 500 to 4000 cm^−1^; scan number: 64 times.

Differential scanning calorimetry (DSC): Used to study the curing behaviors recorded on a DSC Q200 (TA Instruments, New Castle, DE, USA) under nitrogen. Temperature range: 0−300 °C; heating rate: 10 °C min^−1^.

Rheological measurements: To study the polymerization properties of the resin, the rheological behavior was recorded on modular compact rheometer MCR302 from Anton Paar. The sample was placed between disposable plates with 0.5 mm gap, and the experiment was carried out with a temperature ramp mode. Temperature range: 70–250 °C; heating rate: 10 °C min^−1^; frequency: 1 Hz.

Dynamic mechanical analysis (DMA): Thermomechanical properties of the samples were established on DMA Q800 (TA Instruments, New Castle, DE, USA). Temperature range: 25–300 °C; heating rate: 3 °C min^−1^; frequency: 1 Hz.

Thermogravimetric analysis (TGA): TGA Q500 device from TA Instruments was used to study the thermal degradation of the cured samples under nitrogen. Temperature range: 25–800 °C; heating rate: 10 °C min^−1^; frequency: 1 Hz.

### 2.3. T–Fa Preparation and Characterization 

**Scheme 1:**
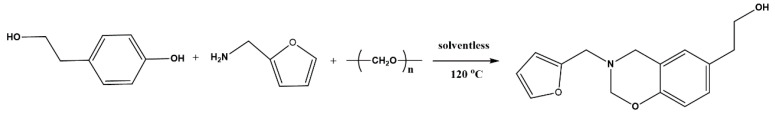
Synthesis of benzoxazine monomer T–Fa.

The synthesis procedure of T–Fa is depicted in Scheme 1, which is similar to the reported method by Ishida et al. [26] More precisely, 20 g (0.145 mol) tyrosol and 14 g (0.145 mol) furfurylamine were mixed in a 250 mL beaker with a mechanical agitator at 120 °C. After mixture dissolution, 9.55 g (0.319 mol) of excess paraformaldehyde was added, and the reaction was maintained for 25 min under continuous stirring. The crude product was dissolved in 200 mL chloroform, and washed with saturated solution of sodium bicarbonate (3 × 200 mL) and deionized water (200 mL) in a separatory funnel. The organic part was collected and concentrated under reduced pressure. Lastly, a white solid was obtained after drying under vacuum at 40 °C overnight (34 g, weight yield 90%).

### 2.4. Curing of T-Fa Resins

The monomer was placed in a stainless-steel mold (60 × 12 × 3 mm), kept at 140 °C in a vacuum oven to remove the bubble for 10 min, and then polymerized step-by-step curing according to the following procedure: 2 h at 150 °C, 2 h at 180 °C, and 1 h at 200 °C.

## 3. Results and Discussion

### 3.1. Preparation and Characterization of Benzoxazine Monomer T–Fa

The synthetic route for the formation of benzoxazine monomer T–Fa is depicted in Scheme 1. A fully biobased benzoxazine monomer T–Fa is prepared by condensation reactions of tyrosol, paraformaldehyde, and furfurylamine via a solventless synthesis procedure.

**Figure 1:**
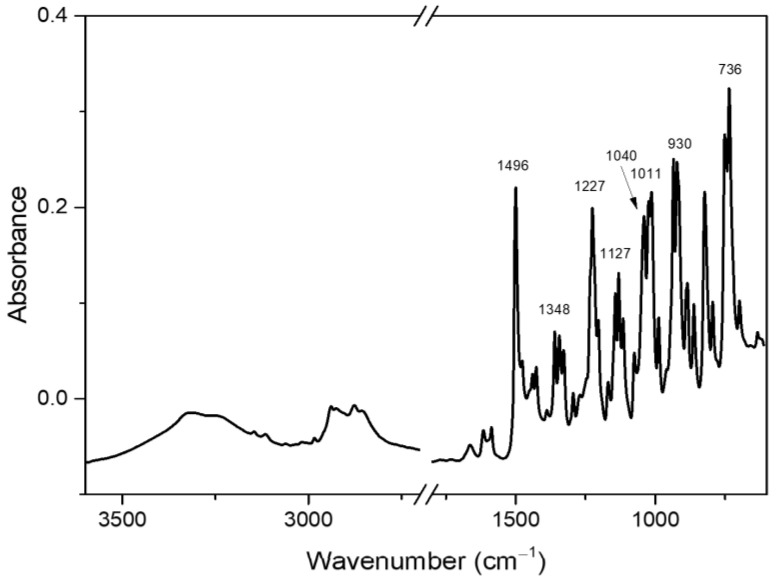
Fourier transform infrared (FTIR) spectrum for T–Fa.

The structure of synthesized benzoxazine monomer was obtained by FTIR. Figure 1 represents the FTIR spectrum of T–Fa. In detail, a broad band at 3250 cm^−1^ corresponded to the stretching vibration bands of the –OH group. Additionally, characteristic absorption bands of the oxazine ring were indicated by the bands at 1496 cm^−1^ (trisubstituted benzene ring stretching), 1342 cm^−1^ (CH_2_ wagging into closed benzoxazine ring), 1227 cm^−1^(aromatic C–O–C asymmetric stretching), 1040 cm^−1^ (asymmetric stretching vibration bands of C–N–C), 1114 cm^−1^ (C–H in plane vibration), and 930 cm^−1^ (C–H out of plane deformation in the aromatic ring fused to the oxazine ring). O–H stretching vibration and out-of-plane wagging deformation were detected at 3327 cm^−1^. Further, a furan ring was detected by characteristic peaks at 1011 and 736 cm^−1^ that were caused by C−O−C antisymmetric stretching and C–H out-of-plane wagging deformation, respectively [19,21].

**Figure 2:**
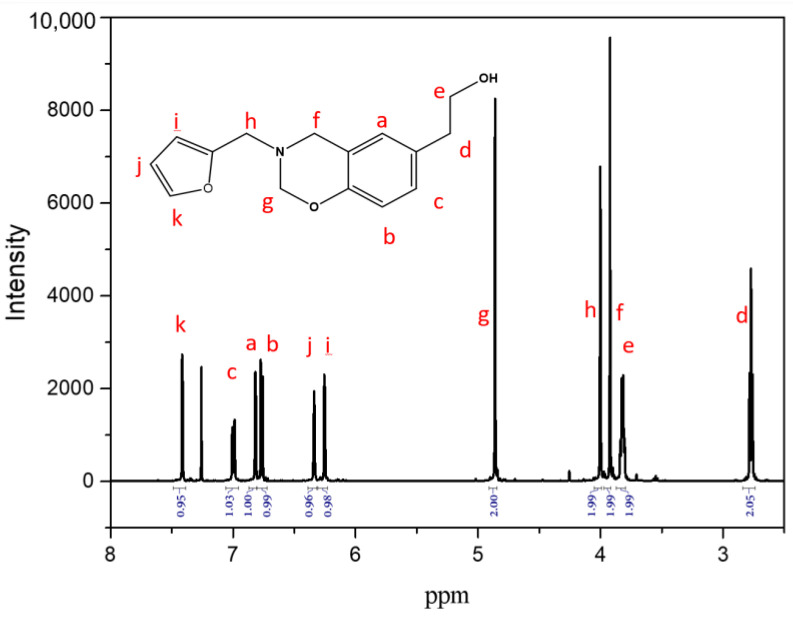
^1^H NMR spectrum of T–Fa in deuterated chloroform (CDCl_3)_.

More detailed structural information of T–Fa was established and confirmed by ^1^H NMR analysis (Figure 2). More precisely, the peaks at 4.85 ppm (δH^g^) and 3.93 ppm (δH^f^) corresponded to the characteristic protons of O–CH_2_–N and Ar–CH_2_–N of the oxazine ring verifying the formation of benzoxazine. The appearance of peaks at δ = 6.81, 6.76, and 6.99 ppm was assigned to aromatic protons close to the benzoxazine ring (δH^a^, δH^b^, and δH^c^). Moreover, the peak of protons from the methylene group was detected at 4.0 ppm (δH^h^), and the characteristic protons of the furan ring appeared at 6.25 (δH^i^), 6.34 (δH^j^), and 7.41 ppm (δH^k^), respectively [6,17]. NMR and FTIR analyses suggested that the desired benzoxazine monomer T–Fa was successfully synthesized.

### 3.2. Curing Behavior of Biobased T–Fa

**Figure 3:**
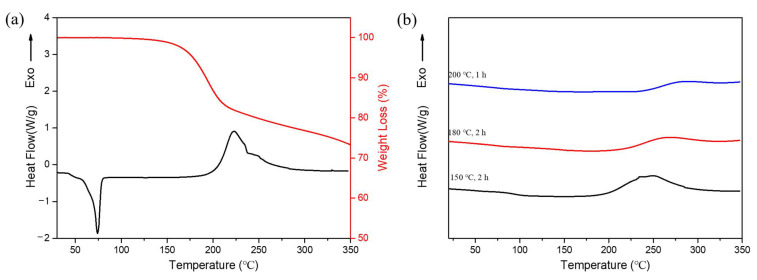
(**a**) Overlay of differential scanning calorimetry (DSC) and TGA–N_2_ heating curves of T–Fa precursor. (**b**) DSC heating curves of p(T–Fa) as recorded after step-by-step curing (after being cured at 150 °C for 2 h, 180 °C for 2 h, and 200 °C for 1 h).

The curing behavior of T–Fa was characterized by DSC (Figure 3a). First, a sharp endothermic peak was observed around at 72 °C. It was assigned to the melting of the T–Fa precursors during the heating scan. Compared to reported furan-based monobenzoxazine monomers [17,24,25,27,28], the melting point of T–Fa was lower than that of most, indicating that our resin had good processability at a relatively low temperature. In addition, two overlapping exothermic peaks with maximal temperatures at 223 and 248 °C were observed with heating, which may have belonged to the ring-opening polymerization of benzoxazine and the additional reaction of the furan group, respectively. As expected, due to the presence of *p*-substituted hydroxyl, T–fa exhibited a lower *T*_p_ at 223 °C than that of the monobenzoxazine P–Fa without hydroxyl, and synthesized with phenol and furfurylamine (*T*_p_ = 241 °C). [23] The value of the exothermic enthalpy of T–Fa was about 254 J g^−1^, indicating good polymerization ability. These results were slightly moderated by TGA data. Indeed, TGA results revealed that a partial degradation of T–Fa occurred in the same range of temperature as that of its polymerization. This phenomenon is often observed and is attributed to the rearrangement of a few reaction intermediates into volatile imine species. The effect is usually more pronounced in the case of monobenzoxazines than that in the case of dibenzoxazines because of the stability of the intermediates. In our case, we observed a weight loss of about 20–25%, as recorded by TGA (Figure 3a). To avoid weight loss during the first stage, we chose 150 °C as the starting ring-opening polymerization temperature.

**Figure 4:**
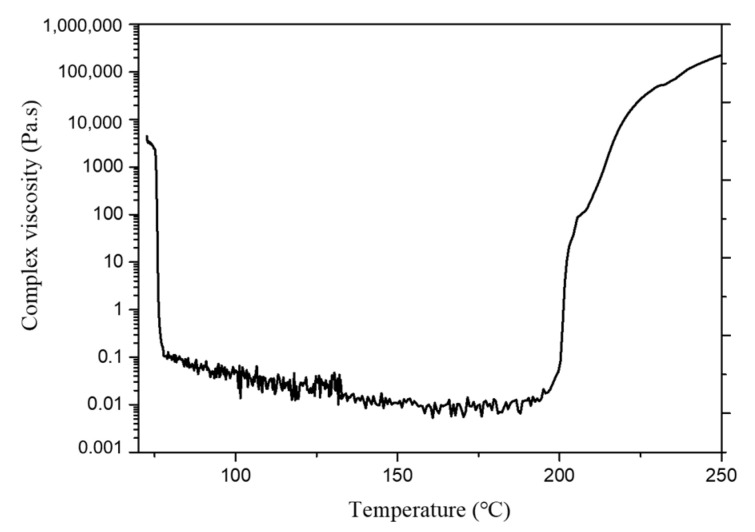
Temperature ramp rheology measurement of T–Fa.

To further determine the polymerization ability of T–Fa and confirm its processing window, the fluidity of the precursors was evaluated by rheology dynamic analysis. The dynamic viscosity profile with a function of temperature is shown in Figure 4. Below 70 °C, T–Fa had a high viscosity platform attributed to its crystallinity. At around 70 °C, viscosity dropped dramatically down to 0.1 Pa.s, and then 0.01 Pa.s as temperature increased to the melting point of the precursors at ~75 °C, confirming the DSC results. At temperatures higher than 200 °C, viscosity increased exponentially due to the polymerization and crosslinking of the resin. Interestingly, the viscosity of the resin remained almost constant in a liquid state in the temperature range of 75–200 °C. This highlights that the low viscosity of the resin at a low temperature (75 °C) with a large temperature range (75–200 °C) exhibited excellent processability on and possibility of coating application.

As shown in Figure 3b, DSC was employed to further study the polymerization process with a step-by-step curing procedure. The exothermic peak and relative enthalpy gradually decreased, meaning the consumption of benzoxazine and furan rings. Lastly, the almost gone exothermic peak indicates that polymerization was completed after curing at 200 °C for 1 h.

**Figure 5:**
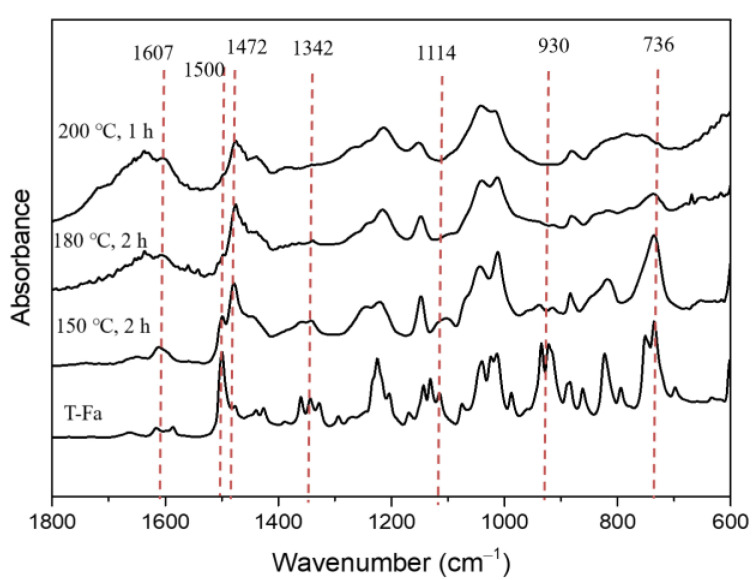
FTIR spectra of T–Fa after step-by-step curing stage (after being cured at 150 °C for 2 h, 180 °C for 2 h, and 200 °C for 1 h).

FTIR technology is often used to monitor the structure evolution and analyze the curing behaviors of benzoxazines (Figure 5). When it was cured step by step, characteristic FTIR signals were associated with the oxazine ring at 930 and 1342 cm^−1^ almost disappeared, witnessing the ring-opening reaction of the benzoxazine monomer. Meanwhile, the characteristic peak of the C–H bond attached to the aromatic structure disappeared at 1114 cm^−1^, confirming the formation of Mannich-type bridge structures. Absorption bands at 1607 cm^−1^ dramatically increased, and the characteristic bands of the substituted benzene ring shifted from 1500 cm^−1^ to lower wave numbers (1472 cm^−1^), indicating a variation in the degree of substitution of the benzene ring. In addition, the characteristic peak at 736 cm^−1^ standing for the furan group disappeared step by step, confirming its involvement in the crosslinking reaction. This result showed that the additional furan crosslinking reaction occurred after the benzene ring-opening reaction.

### 3.3. Properties of Biobased Cured p(T-Fa)

**Figure 6:**
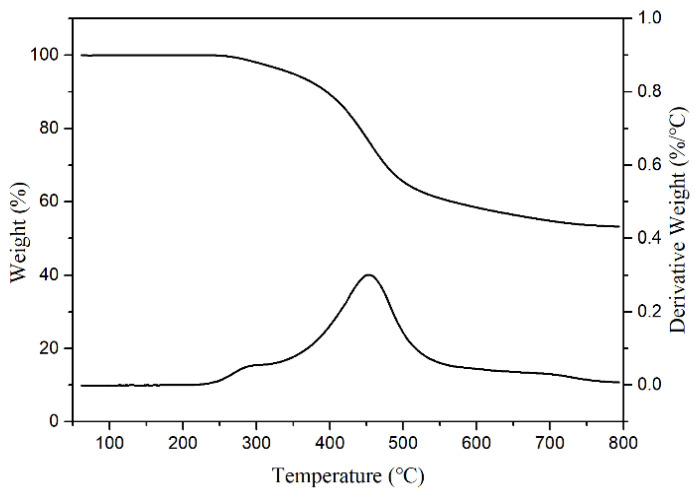
TGA curves for p(T–Fa) under nitrogen.

The thermal stability of p(T-Fa) was evaluated by TGA, as shown in Figure 6. With furfurylamine participating in polymerization, this monobenzoxazine T–Fa formed a crosslinking structure to enhance stability. After curing, p(T–Fa) underwent two main stages of degradation. The first was observed in the range of 250–322 °C. The almost negligible presence might be explained by the loss of small oligomers resulting from secondary reactions upon curing that remained trapped in the crosslinked network. Second, a major weight-loss rate was clearly detected in the range of 322–550 °C associated with the main chain degradation of the resin. Moreover, 5% and 10% weight loss (*T*_d5_ and *T*_d10_) of p(T–Fa) were obtained at 349 and 395 °C, respectively. Lastly, residual weight at 800 °C (char yield) was as high as 53%. These relevant results exemplified the excellent thermal stability of p(T–Fa).

**Figure 7:**
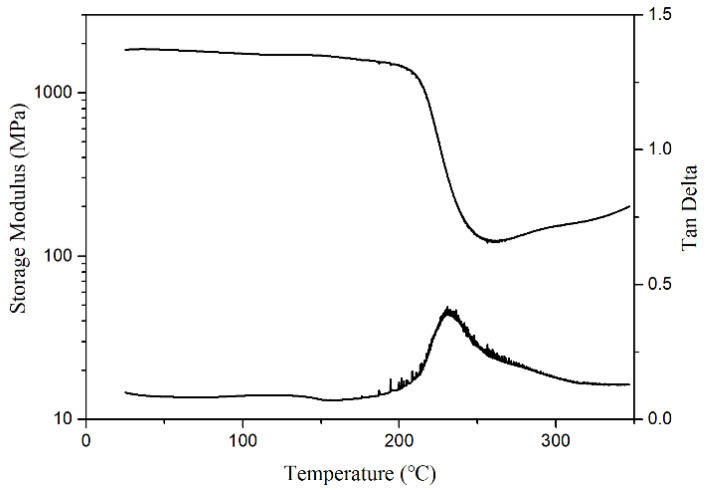
Thermomechanical behavior of p(T–Fa) by dynamic mechanical thermal (DMA) analysis.

The determination of the glass-transition temperature (*T*_g_) of the cured p(T–Fa) system was performed by DSC, although detection was uneasy, as shown in Figure 3b. The p(T–Fa) resin presented quite a high *T*_g_ of about 217 °C. Moreover, the thermomechanical transition associated with the glass transition was evaluated using DMA. As shown in Figure 7, it appeared that the cured p(T–Fa) exhibited a high *T*_g_ at 232 °C, and a high storage modulus G_0_ was maintained in the glassy state (1.8 GPa). The high value of *T*_g_ agreed with DSC results. Making a comparison with other related biobased monobenzoxazines, the p(T–Fa) resin demonstrated good and competitive performance.

## 4. Conclusions

A new, potentially fully biobased monobenzoxazine T–Fa starting from tyrosol and furfurylamine was successfully synthesized that can be both elaborated from renewable resources. The precursor presented a low melting temperature around 70 °C, as observed by DSC, and complex viscosity as low as 0.01 Pa.s, as determined by rheology dynamic analysis. The valuable large processing window allowed for us to consider solventless applicability and the use of such a resin. In addition, the polymerization ability of the precursor was well-evidenced by DSC and rheology measurements. FTIR results revealed that thermal polymerization proceeded via the ring-opening process of the benzoxazine rings and additional crosslinking of the furan functions accelerated by hydroxyl functions. After curing, a crosslinked network of p(T–Fa) exhibiting high thermal and thermomechanical properties was achieved, highlighting a *T*_g_ of 232 °C, *T*_d5_ of 349 °C, *T*_d10_ of 395 °C, and a char yield of 53%. The combination of these characteristics and properties made this fully biobased product a promising candidate for anticorrosion-coating applications.

## Data Availability

The data that support the findings of this study are contained within the article.
